# Mental Health in the Shadow of Conflict: Psychological Profiles and Pathways to Suicidal Ideation in Conflict-Affected Students

**DOI:** 10.3390/ejihpe15110232

**Published:** 2025-11-13

**Authors:** Sami Hamdan, Eyad Hallaq

**Affiliations:** 1 School of Behavioral Sciences, Academic College of Tel-Aviv Jaffa (MTA), P.O. Box 8401, Tel-Aviv-Jaffa 61083, Israel; 2 Department of Psychology, Al-Quds University, Abu Dis P144, Palestine; ihalaq@staff.alquds.edu

**Keywords:** Palestinian youth, suicidal ideation, mental health, conflict trauma, self-efficacy, resilience, latent profiles

## Abstract

**Objective:** This study aimed to identify psychological characteristics associated with suicidal ideation among Palestinian university students in the West Bank during a period of escalating regional violence (October 2023), with data collected prior to the end of the war, a period marked by intensified political violence and collective trauma. The goal was to identify empirically derived psychological profiles of distress and coping using Latent Profile Analysis. **Method:** A cross-sectional survey of 900 students assessed depression, anxiety, self-efficacy, resilience, help-seeking attitudes, and suicidal ideation during the past 12 months. Latent Profile Analysis (LPA), logistic regression, and moderated mediation analysis were employed to investigate the relationships between distress, self-efficacy, resilience, and suicidal ideation. **Results:** Results indicate that depression and anxiety are associated with increased 12-month suicidal ideation, but greater self-efficacy appears to reduce this risk. The mediation analysis revealed that self-efficacy partially explains the relationship between distress and suicidal ideation; however, resilience did not have a significant moderating effect. The LPA identified three distinct psychological profiles, with the highest-risk group exhibiting significant distress and low self-efficacy. **Conclusions:** These results highlight the significant mental health burden faced by Palestinian youth and underscore the importance of internal psychological resources, particularly self-efficacy, that are associated with lower levels of suicidal ideation. Enhancing self-efficacy may offer a culturally relevant approach for prevention efforts in politically unstable environments.

## 1. Introduction

Transitioning to university is a significant life stage with higher academic demands, identity formation, and personal responsibilities. This developmental period has been identified as the most vulnerable to psychological vulnerability across various cultural contexts, with students bearing the greatest burden in terms of the incidence of psychological disorders such as depression, anxiety, and suicidal ideation (Eisenberg et al., 2013).

Among university students in regions affected by political conflict, such as the West Bank and Gaza Strip, these stressors interact with structural violence, economic uncertainty, and ongoing engagement with collective trauma, combining to worsen psychological distress and disrupt routine routes to psychological support (Hamdan & Hallaq, 2021). Research has consistently demonstrated high rates of psychological distress and suicidal ideation and behaviors, shaped by decades of political volatility, mobility limitations, and systemic barriers to care (Giacaman et al., 2011; Hamdan & Hallaq, 2021; Mahamid et al., 2024). Hamdan and colleagues (Hamdan & Hallaq, 2021) found that prolonged exposure to violence predicts depressive symptoms and suicide risk among Palestinian students, while others have highlighted the mediating roles of social support, spirituality, and emotional resilience (Mahamid et al., 2023).

To understand suicidal ideation in this population, it is essential to situate mental health outcomes within the broader framework of social determinants (including political violence, systemic marginalization, and constrained health infrastructure) that uniquely shape lived experience in conflict zones. Furthermore, the psychological sequelae of conflict among Palestinian youth cannot be adequately understood without considering the embedded sociocultural and political structures that shape daily life (Giacaman et al., 2011). Sociocultural factors such as family obligation, communal identity, and intergenerational trauma complicate how psychological constructs manifest in collectivist settings. Within a collectivist context, individual distress is often mediated through familial obligations, intergenerational narratives, and communal forms of belonging, which collectively shape both vulnerability and resilience (Mahamid et al., 2024). Empirical studies among Palestinian youth show that communal coping predicts better emotional outcomes under chronic conflict (Veronese et al., 2020). These dynamics, compounded by structural violence and weakened civic infrastructure, create a psychosocial climate in which Western psychological constructs may not fully capture culturally embedded expressions of resilience and distress (Nguyen-Gillham et al., 2008).

In this context, the current study examined three psychological variables that may play a protective or mediating role. Self-efficacy is a mediating internal resource that influences how individuals process and respond to distress. Operationally, it was measured as the perceived confidence in managing academic, social, and emotional demands (Schwarzer & Jerusalem, 1995). Bandura’s social cognitive theory (Bandura, 1997) underscores the centrality of self-efficacy in shaping behavior under stress, suggesting that individuals with higher self-efficacy are better able to regulate emotions, maintain motivation, and cope with adversity. Empirical evidence supports this mediating role, showing that self-efficacy links depression and anxiety to suicidal ideation through adaptive coping and emotional regulation (Liu et al., 2023).

Resilience was examined as a moderating variable that may buffer indirect effects of distress in suicidal ideation. These roles are supported by various studies suggesting that internal psychological strengths can shape how an individual processes trauma (Arango et al., 2023; Mitchell et al., 2020; Wattick et al., 2023). The protective mental factors of psychological resilience and self-efficacy help people reduce their vulnerability to traumatic events and psychological distress (Mitchell et al., 2020).

Finally, attitudes toward seeking professional psychological help were assessed to capture culturally relevant patterns of support utilization. In collectivist and conflict-affected societies, stigma, fatalism, and family dynamics influence whether distress leads to professional help-seeking. Positive attitudes can mitigate emotional distress by facilitating access to formal psychological resources (Lazarus, 1993; Vogel & Wei, 2005). In stigmatized or resource-poor, conflict-affected areas, negative help-seeking attitudes can delay intervention and increase risk (El Khoury-Malhame et al., 2025; Lu et al., 2025).

Together, these constructs form an integrated stress-coping framework in which psychological distress affects suicidal ideation both directly and indirectly through self-efficacy, while resilience moderates this pathway and help-seeking attitudes reflect culturally specific behavioral responses to distress.

The current study was conducted in the context of the 7 October 2023 military escalation in southern Israel and the subsequent conflict in Gaza. This conflict had significant psychological effects that extended to West Bank university students, who were indirectly exposed through media saturation, university closures, and generalized social disruption. Recent empirical studies have documented substantial increases in anxiety, depression, and post-traumatic symptoms among Israeli and Palestinian students following the 7 October events (Dopelt & Houminer-Klepar, 2024; Groweiss et al., 2024).

While prior studies have identified risk factors for suicide in conflict-affected populations, fewer have explored the mechanisms by which these risks are amplified or mitigated. Building on this gap, the present study tests a mediation-moderation model in which self-efficacy mediates, and resilience moderates, the relationship between psychological distress and suicidal ideation. Latent Profile Analysis was used to explore heterogeneity in students’ mental health responses, identifying groups that share similar patterns of distress and coping.

The proposed moderated mediation model assumes that psychological distress (depression and anxiety) increases suicidal ideation both directly and indirectly through lower self-efficacy. Resilience is expected to moderate this indirect effect, such that higher resilience reduces the negative influence of distress on self-efficacy and suicidal ideation

Specifically, we hypothesized that: (1) higher levels of depression and anxiety would be associated with increased suicidal ideation; (2) self-efficacy would mediate the relationship between distress and suicidal thoughts; and (3) resilience would moderate the indirect path such that higher resilience would buffer the effect of distress via self-efficacy. This study aims to contribute to the global literature on the social and psychological determinants of suicide risk. These hypotheses outline the main pathways of the proposed stress-coping model, while acknowledging that additional sociocultural factors may also play a role. Attitudes toward help-seeking were analyzed descriptively as a culturally relevant factor affecting support utilization.

## 2. Methods

### 2.1. Participants and Sampling Procedure

The sample consisted of 900 Palestinian undergraduate students enrolled at several West Bank universities during the 2024–2025 academic year. Participants were recruited using an online convenience sampling method, with survey links distributed primarily through student-oriented social media networks and groups frequented by Palestinian university students, such as Facebook and WhatsApp groups affiliated with universities, academic programs, or student organizations. The online survey was administered via Qualtrics between February 2024 and January 2025, several months after the 7 October 2023 escalation. Before completing the survey, electronic informed consent was obtained, and participation was entirely voluntary, confidential, and without any incentives or compensation. The study was approved by the Institutional Review Board (IRB) of the Academic College of Tel Aviv Jaffa and Al-Quds University. Participants were provided with a list of local and national mental health support services at the conclusion of the questionnaire to ensure access to appropriate care if needed.

A priori power analysis was used to calculate the target sample size using G*Power software V. 3.1 (Faul et al., 2007). For logistic regression investigations with up to 10 variables, a medium predicted effect size (odds ratio ≈ 1.5), α = 0.05, and power = 0.80, the minimum required sample size was around 750 students. To account for any incomplete responses or missing data, the figure was reduced by approximately 20%, resulting in a target sample size of around 900 individuals.

A total of 1153 students initiated the survey, and 900 of them completed it, resulting in a completion rate of approximately 78%. Demographically, the Participants’ gender distribution closely mirrored that of the total Palestinian university student population (about 60% female), as reported by the Palestinian Ministry of Higher Education and Scientific Research (Palestinian Ministry of Higher Education and Scientific Research, 2023). Furthermore, participants represented a range of academic fields consistent with national enrollment patterns.

The majority of participants were female (*n* = 550, 61.1%) and unmarried (*n* = 600, 66.7%). The sample represented various academic fields, including social sciences (*n* = 230, 25.6%), humanities (*n* = 210, 23.3%), and natural sciences (*n* = 180, 20.0%). The average age of the participants was 24.91 years (SD = 6.39).

### 2.2. Measures

Participants completed a battery of standardized self-report questionnaires that measured psychological distress, protective variables, and attitudes toward help-seeking and suicidal conduct. All instruments were administered in Arabic using verified translations or according to the best standards for cross-cultural research (Beaton et al., 2000).

**Suicidal Ideation:** Suicidal ideation during the past 12 months was assessed using a single dichotomous item adapted conceptually from the Columbia–Suicide Severity Rating Scale C-SSRS; (Posner et al., 2011). The item was based on the “Wish to Be Dead” and “Non-Specific Active Suicidal Thoughts” categories of the C-SSRS, which include passive and general suicidal thoughts. Participants were asked: “During the past 12 months, have you had thoughts of wishing you were dead or of wanting to end your life?” (0 = No, 1 = Yes). This simplified format was chosen to reduce participant burden while maintaining conceptual alignment with validated C-SSRS domains. Although single-item questions may risk some misclassification, similar measures have demonstrated adequate validity and reliability in population-based research (Millner et al., 2015). Although the use of a single dichotomous item may limit sensitivity to gradations of suicidal ideation, this approach minimizes participant burden and is consistent with large-scale epidemiological studies (Hamdan & Hallaq, 2021). Furthermore, the observed 8.8% 12-month suicidal ideation rate is consistent with prior research among Palestinian university students and other studies in neighboring Middle Eastern contexts (Arafat et al., 2023), supporting the validity of this estimate within a culturally sensitive framework.

**Depression:** Depressive symptoms were measured using the Patient Health Questionnaire-9 PHQ-9; (Kroenke et al., 2001), a widely used 9-item scale assessing the frequency of depressive symptoms over the past two weeks. Responses range from 0 (not at all) to 3 (nearly every day), with total scores ranging from 0 to 27. Higher scores indicate more significant depressive symptomatology. Internal consistency in the current sample was excellent (α = 0.89).

**Anxiety:** Anxiety symptoms were assessed using the Generalized Anxiety Disorder 7-item scale GAD-7; (Spitzer et al., 2006). Respondents rated how often they experienced anxiety-related symptoms over the past two weeks on a 4-point scale (0 = not at all to 3 = nearly every day). Total scores range from 0 to 21, with higher scores indicating greater severity of anxiety. Cronbach’s alpha in the present sample was 0.91.

**General Self-Efficacy:** Self-efficacy was measured using the General Self-Efficacy Scale GSE; (Schwarzer & Jerusalem, 1995), a 10-item scale assessing an individual’s perceived ability to cope with daily challenges and stressful situations. Items are rated on a 4-point scale from 1 (not at all true) to 4 (exactly true). Higher scores reflect stronger self-efficacy. Internal consistency in the current study was α = 0.87.

**Resilience:** The Brief Resilience Scale (BRS; (Smith et al., 2008) was used to assess the ability to recover from stress. The BRS includes 6 items rated on a 5-point scale (1 = strongly disagree to 5 = strongly agree), with some items reverse-coded. Higher total scores indicate greater resilience. Cronbach’s alpha in this sample was 0.83.

**Attitudes Toward Seeking Professional Psychological Help:** Participants’ help-seeking attitudes were assessed using the Attitudes Toward Seeking Professional Psychological Help—Short Form ATSPPH-SF; (Fischer & Farina, 1995). This 10-item scale uses a 4-point Likert scale (0 = disagree to 3 = agree) to assess openness to psychological help. Higher scores indicate more positive attitudes toward seeking help. Internal consistency in this study was acceptable (α = 0.79).

Sociodemographic and academic variables included, age (in years), marital status (single/married/other), year of study (1st through 4th year), and academic discipline (e.g., health sciences, engineering, social sciences). Gender (biological sex assigned at birth) was coded as 0 = male, 1 = female. These were assessed via self-report and included in descriptive analyses. Gender was included as a covariate in multivariate analyses due to theoretical and empirical relevance to suicidal ideation.

### 2.3. Ethics Statement

The study was approved by the Institutional Review Board of the Academic College of Tel-Aviv Jaffa (MTA) (MTA-IRB/PSY-2024-011) and the Ethics Committee of Al-Quds University (AQ-PSY-24/IRB-004). All participants provided informed consent before starting the survey. The consent form explicitly stated that some questions would address sensitive topics, including suicidal thoughts and emotional distress, and that participants could withdraw at any time without penalty. At the end of the survey, participants received contact information for local mental-health services and university counseling centers. No further intervention or real-time monitoring was conducted.

### 2.4. Data Analysis

Data analysis was carried out using SPSS (Version 29; IBM Corp., 2022, Armonk, NY, USA), the PROCESS macro for SPSS (Hayes, 2013), and Mplus. Missing data (3.9%) were addressed through multiple imputations using fully conditional specification, and analyses were conducted on pooled datasets.

Descriptive statistics summarize demographic and psychological variables. Group differences between participants with and without suicidal ideation were tested using independent *t*-tests and chi-square analyses. For both depression and anxiety, continuous total scores were used in regression analyses and latent profile analyses (LPA) to capture the full range of symptom severity. Categorical thresholds (e.g., mild, moderate, severe) were employed solely for descriptive reporting in the sample characteristics.

A binary logistic regression model was used to identify predictors of suicidal ideation, including depression, anxiety, self-efficacy, resilience, and help-seeking attitudes, with gender included as a covariate.

The conceptual model tests whether psychological distress (depression and anxiety) influences suicidal ideation directly and indirectly through self-efficacy, and whether this indirect effect is moderated by resilience. PROCESS Model 14 was applied with 5000 bootstrap samples to estimate conditional indirect effects. Gender was included as a covariate based on prior evidence of sex differences in suicidality.

Latent Profile Analysis (LPA), a person-centered approach (Collins & Lanza, 2009), was performed to identify subgroups of students with similar patterns of distress and coping, based on scores for depression, anxiety, self-efficacy, resilience, and help-seeking attitudes. Model fit was assessed using the Bayesian Information Criterion (BIC), Akaike Information Criterion (AIC), entropy, and the Lo–Mendell–Rubin likelihood ratio test. The optimal model was selected based on fit indices and interpretability, and profile differences in suicidal ideation were analyzed using one-way ANOVA. This analysis directly supports the study’s goal of identifying empirically derived psychological risk profiles within a conflict-affected student population (Zerach et al., 2019).

All statistical tests had a significance level of *p* < 0.05. Confidence intervals and effect sizes were also reported.

## 3. Results

As presented in Table 1, in terms of clinical features of the sample, 8.8% (*n* = 79) reported experiencing suicidal ideation in the past year. Additionally, moderate to severe symptoms of depression and anxiety were reported by 42.3% and 33.9% of the sample, respectively.

Table 1 summarizes the demographic and clinical characteristics of the sample.

As shown in Table 2, students who reported 12-month suicidal ideation scored significantly higher in depression, t(897) = −4.18, *p* < 0.001, and anxiety, t(897) = −6.67, *p* < 0.001, than those without such thoughts. In contrast, students without suicidal ideation exhibited significantly higher levels of resilience, t(897) = 3.35, *p* < 0.001, and self-efficacy, t(897) = 5.71, *p* < 0.001, suggesting these traits may provide a protective role. A modest but statistically significant difference was also observed in help-seeking attitudes, t(897) = 3.91, *p* = 0.002.

No significant group differences were found based on academic discipline, χ^2^(4) = 1.48, *p* = 0.831, or marital status, χ^2^(3) = 3.98, *p* = 0.264. Gender differences approached statistical significance, χ^2^(1) = 4.00, *p* = 0.055, with a higher proportion of females in the non-suicidal group.

A binary logistic regression analysis was conducted to examine whether depression, anxiety, resilience, self-efficacy, help-seeking attitudes, age, and gender were associated with the likelihood of reporting suicidal ideation in the past 12 months. The overall model was statistically significant, χ^2^(7) = 45.32, *p* < 0.001, suggesting that the combination of variables successfully differentiated between students who reported suicidal ideation and those who did not. The model accounted for approximately 14% of the variance in suicidal ideation, as indicated by the Nagelkerke R^2^, and demonstrated acceptable fit to the data (Hosmer–Lemeshow χ^2^(8) = 6.72, *p* = 0.57). As shown in Table 3, higher levels of depression (OR = 1.07, *p* = 0.004) and anxiety (OR = 1.11, *p* = 0.001) were significantly associated with increased odds of suicidal behaviors. Conversely, greater self-efficacy was associated with decreased odds of suicidal behavior (OR = 0.94, *p* = 0.001). Resilience, help-seeking attitudes, age, and gender were not statistically significant predictors.

In the depression model, depression significantly predicted suicidal ideation both directly (B = 0.038, *p* < 0.001) and indirectly through lower self-efficacy (indirect effect = 0.003, SE = 0.001, 95% CI [0.001, 0.005], *p* = 0.002). The index of moderated mediation was non-significant (0.0001, 95% CI [−0.0001, 0.0003]), indicating that the indirect effect via self-efficacy did not vary significantly across levels of resilience.

A similar pattern was found in the anxiety model. Anxiety had a significant direct effect on suicidal ideation (B = 0.039, SE = 0.005, 95% CI [0.029, 0.048], *p* < 0.001) and an indirect effect through self-efficacy (indirect effect = 0.0036, SE = 0.0014, 95% CI [0.0012, 0.0066], *p* = 0.002). Again, the moderated mediation index was not significant (0.0001, 95% CI [−0.0001, 0.0003]).

These findings suggest that while self-efficacy partially mediates the relationship between psychological distress and suicidal ideation, this mediation pathway is not significantly influenced by the level of resilience.

To examine whether the protective role of self-efficacy varied according to participants’ levels of resilience, we tested a moderated mediation model (PROCESS Model 14). The statistical index of moderated mediation was non-significant in both cases for depression (B = −0.001, 95% CI = [−0.003, 0.0004]) and for anxiety (B = −0.001, 95% CI = [−0.002, 0.0004]). In other words, the strength of the indirect effect from psychological distress to suicidal ideation through self-efficacy did not depend on how resilient the participants were. This suggests that resilience, as measured in this study, did not significantly alter the impact of self-efficacy on suicidal ideation. Detailed results are presented in Table 4.

The hypothesized moderating role of resilience was not supported. The interaction term between resilience and self-efficacy in relation to suicidal ideation was nonsignificant (*p* = 0.37). This indicates that the relationship between self-efficacy and suicidal ideation remained consistent across different levels of resilience. In this sample, resilience did not seem to influence the link between psychological distress and suicidal ideation.

The buffering role of resilience, which was hypothesized to reduce the risk of suicidal ideation at higher levels of psychological distress, was not statistically supported. This lack of moderation indicates that the protective effect of self-efficacy on suicidal ideation remains stable regardless of individuals’ levels of resilience. This implies that, within this sample and cultural context, resilience may not exert a buffering effect beyond that provided by self-efficacy.

**Figure 1:**
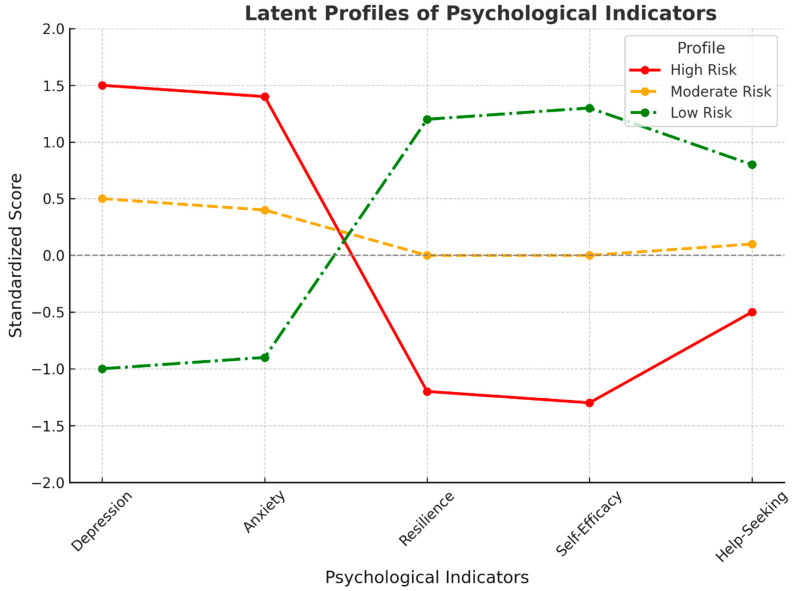
Latent Profiles of Psychological Indicators. **Note.** Standardized mean scores are presented for each psychological indicator across three latent profiles identified through covariate-adjusted latent profile analysis. Gender was included as a covariate. Higher values reflect a greater endorsement of each construct. The profiles represent distinct psychological patterns labeled as High Risk, Moderate Risk, and Low Risk.

Latent Profile Analysis (LPA) was performed to identify homogeneous subgroups of students characterized by different patterns of distress and protective factors. A three-profile solution showed the best fit (BIC = 10,744.10; entropy = 0.83), effectively balancing statistical accuracy and clinical relevance interpretability. Figure 1 illustrates the standardized mean scores of depression, anxiety, resilience, self-efficacy, and help-seeking attitudes across the three latent profiles, which included gender as a covariate. The **High-Risk** profile (23.8% of the sample) was characterized by elevated levels of depression and anxiety and markedly low levels of self-efficacy and resilience. The **Moderate Risk group** (41.2%) displayed average scores across all indicators. **The Low-Risk** profile (35%) showed low psychological distress and high levels of protective factors.

A one-way ANOVA revealed significant differences in suicidal ideation across the three profiles, F(2, 897) = 24.32, *p* < 0.001. Post hoc comparisons indicated that individuals in the High-Risk group reported significantly higher levels of suicidal ideation than those in both the Moderate and Low-Risk profiles.

## 4. Discussion

This study examined psychological characteristics associated with 12-month suicidal ideation among Palestinian university students in the West Bank during the 2024–2025 academic year, a period of increased social and political unrest following the 7 October Hamas mass attack and subsequent Israel-Hamas War. By integrating variable-centered (moderated mediation) and person-centered (latent profile) approaches, this study provides a comprehensive perspective on both the mechanisms and the heterogeneity of suicide risk among conflict-affected students. For consistency, all analyses and references to suicidal ideation in this manuscript refer to ideation reported within the past 12 months. Higher levels of depression and anxiety were correlated with a higher likelihood of suicidal ideation, the results showed. Conversely, participants with higher self-efficacy reported significantly lower suicidal ideation, suggesting that confidence in one’s coping ability functions as a psychological buffer. While resilience was considered a psychological buffer in times of adversity, it did not significantly mediate the relationship between distress (depression and anxiety) and suicidal ideation in the present sample. These findings shed light on the mental health landscape of Palestinian youth living under chronic conflict and illuminate not just the prevalence of psychological distress in Palestinian youth but also the internal psychological mechanisms that can increase and decrease suicide risk.

This finding is consistent with the established research literature, which links emotional dysregulation, hopelessness, and feelings of burdensomeness to suicidality, particularly among conflict-affected populations (Levi-Belz et al., 2024). In the Palestinian case, this affective disorganization is more than a localized intrapsychic phenomenon; it translates broader political, economic, and social conditions into communal effects. This underscores that individual distress cannot be separated from sociopolitical context; Palestinian students experience mental health not solely as personal suffering but as part of a collective response to ongoing occupation and instability (Giacaman et al., 2011; Nguyen-Gillham et al., 2008) It is this that these young adults are facing, in addition to academic pressures, employment precarity, and ambiguous life trajectories that are prolonged by displacement and violence, compounded by structural violence and mobility restrictions that exacerbate personal trauma and distress. In accord with previous evidence (Hamdan & Hallaq, 2021), the high prevalence rates of depression and anxiety identified in the current study were probably attributed to these domains of compounded adversity. In particular, the mediating role of self-efficacy in the relationship between psychological distress and suicidal ideation. Self-efficacy—an individual’s perception that they can handle a threat and subsequently manage the outcome—stands out as a key psychological buffer. Higher levels of self-efficacy in students are associated with having lower suicidal ideation despite the presence of depressive or anxious symptoms. This finding is consistent with Bandura’s (1997) social cognitive theory, suggesting that people who are confident in their ability to cope more readily engage in adaptive coping and are more resilient to stressful events. In the context of long-standing political instability, when external control may be out of reach, one internal resource of self-efficacy can serve as an alternative domain for perceived agencies. In this way, self-efficacy may also reflect culturally rooted forms of agency, especially among female students managing limited autonomy and among males restricted by social expectations of toughness and control. Future research should explore gender-specific patterns in how efficacy beliefs influence adaptive coping behaviors. Recent studies among Arab and Middle Eastern youth, in line with the aforementioned reservations about self-efficacy, showed that higher self-efficacy was associated with poorer emotional regulation and lower suicidal behavior risk (Mahamid et al., 2021).

Resilience did not show a statistically significant interaction with self-efficacy in relation to suicidal ideation. Although resilience is often described as a protective correlate in trauma-exposed populations, its statistical contribution in this study was minimal. One possible reason is measurement: the Brief Resilience Scale primarily captures individualistic ideas of “bouncing back” from adversity and may not include collective, faith-based, or politically rooted forms of resilience common in Palestinian communities. In contexts characterized by prolonged military violence, displacement, and limited institutional support, such culturally ingrained forms of resilience may serve as a gradual adaptation process rather than an immediate way to reduce distress. Evidence from other collectivist and conflict-affected samples likewise indicates that resilience tends to manifest as a multidimensional construct shaped by social and spiritual resources rather than by individual traits alone (Fino et al., 2020; Veronese et al., 2020). Accordingly, future research could benefit from examining culturally adapted or multidimensional measures of resilience to capture these broader dimensions.

Latent profile analysis identified three psychological subgroups: low risk, moderate risk, and high risk. High-risk students, based on the severity of depression, anxiety, low self-efficacy, and resilience, were more likely to exhibit suicidal ideation. This person-oriented view brings out the heterogeneity of the student population and the need for personalized intervention strategies. Universal mental health care may not be adequate for the high-risk group unless it contains components that buffer hopelessness and facilitate cognitive self-regulation. Indeed, students with a high-risk profile were nearly six times more likely to report suicidal ideation than those with a low-risk profile, highlighting the need for tiered interventions that match students’ psychological needs to appropriate levels of care (Freeman, 2022).

The timing of the study makes it relevant to the interpretation of the severity of the psychological distress and suicidal ideation in the sample. Data collection began after the outbreak of widespread violence on 7 October 2023, followed by the war. While the physical effects of the war were localized in some areas, its psychological effects spread throughout Palestinian society, including the West Bank and Israel. Throughout the study, the students were subjected to widespread media coverage, widespread bereavement, disruption of academic life, and general anxiety and insecurity about the future, all of which would have caused psychological distress in this group. This contextual sensitivity is critical: it indicates that acute conflict exposure interacts with pre-existing structural adversity to amplify suicidal vulnerability.

These studies add to the mounting body of evidence for the devastating impact of the war on the mental health of young adults. Recent studies attest to rises in symptoms of anxiety, depression, and symptoms of trauma in adolescents and young adults in war areas, especially in the months following the war (Groweiss et al., 2024). The psychological environment during this period was shaped not only by direct or indirect exposure to violence but also by the long-term effects of structural instability, displacement, and collective grief. Under such circumstances, emotional suffering exceeds the model of personal vulnerability; it reflects a larger sociopolitical atmosphere in which psychological safety is under constant threat. These studies indicate that the psychological impact of mass violence may be observed across the population in the war areas, though their lived experiences and the sociopolitical atmosphere of the sample are extremely varied. Taken together, the findings show a pattern where internal psychological resources (especially self-efficacy) mediate but do not eliminate the harmful effects of structural and political stressors. This emphasizes the need for both individual coping improvements and systemic interventions that address chronic insecurity and lack of resources.

On the contrary, there are some limitations that must be taken into account. Cross-sectional design restricts our ability to make causal inferences regarding the interplay of suicidal ideation, psychological distress, and resilience factors. It would be better to examine changes over time, as well as the dynamic interplay of risk and resilience, in longitudinal studies. Secondly, using self-report questionnaires may confound reporting biases, particularly in stigmatized cultures regarding suicide and mental illness. The online convenience sample strategy may have also excluded underrepresented or underserved groups (e.g., residents of Gaza or rural students), as students without regular internet access or who are disengaged from online academic platforms may be underrepresented. Thirdly, the employed resilience instrument may not have assessed the diverse, culturally embedded nature of resilience in this context. Moreover, help-seeking attitudes were measured at a single point in time; future longitudinal studies should examine whether positive attitudes forecast later use of psychological services. A notable limitation is the assessment of suicidal ideation using a single dichotomous item, which limits the ability to capture gradations of severity and may underestimate prevalence. Future studies should consider using validated multi-item scales that assess frequency, intensity, and duration to better capture the complexity of suicidal thoughts. Lastly, the timing of data collection (shortly after the October 2023 escalation) may have elevated distress levels in the sample. Without pre-war baseline data, it is difficult to disentangle acute conflict-related stress from longer-term psychological effects.

Despite these limitations, the study has different clinical implications. Mental health screening for anxiety, depression, and suicidal ideation must become regularly integrated into university programs, especially where there is greater volatility. Community-level interventions, such as mental health outreach embedded within universities, public education campaigns, and policy reforms targeting student well-being, are essential for addressing the collective dimensions of psychiatric risk in conflict settings. Specific focus should be on early detection and gatekeeper training at universities, integrating mental health liaisons who can identify and refer high-risk students. Digital mental health tools and anonymous chat-based support can further decrease stigma and improve access (Goh et al., 2021). These initiatives should be integrated into broader community mental health strategies that prioritize accessibility, cultural sensitivity, and long-term sustainability in underserved populations. In the Palestinian cultural context, gender norms may also shape mental health experiences and help-seeking behaviors. Evidence from regional studies shows that Arab male students often hesitate to express emotional distress or seek counseling because of masculine norms that emphasize self-control and emotional restraint. (Mahamid et al., 2024; Noorwali et al., 2022). Conversely, female students may experience greater family and community monitoring, which can heighten distress and restrict access to formal psychological support (Abo-Rass, 2024). Addressing these gender-specific barriers is vital for providing culturally responsive and equitable mental health services in university settings.

The study also highlights the importance of therapy that promotes self-efficacy, such as cognitive-behavioral skill training, mentoring, and strengths-based psychoeducation. Since the resilience of the person has its limits in the context of greater adversity, mental health care must be supplemented with social and institutional interventions to enhance the building of community trust and psychological safety. Culturally sensitive, trauma-informed, low-cost treatments, including digital interventions, could be extremely effective in communities where emotional help-seeking continues to remain stigmatized or inaccessible.

Overall, this study makes a contribution to the expanding body of literature regarding mental health and suicidal risk in the context of political violence and chronic instability. Suicidal ideation in Palestinian university students was highly correlated with heightened psychological distress and the availability, or lack thereof, of internal psychological resources in the form of self-efficacy. These findings emphasize the importance of culturally appropriate mental health interventions that are not merely symptom reduction but actively enhance coping ability and psychological resilience. While rooted in the Palestinian context, these insights contribute to global mental health discourse by highlighting how political instability and structural adversity shape psychological risk and resilience in young adults across conflict-affected settings. Importantly, the findings challenge common assumptions about the universal buffering role of resilience, emphasizing the need to contextualize psychological resources within local cultural and structural realities. By showing how self-efficacy stays protective even in widespread instability, the study highlights that encouraging agency and perceived competence can be an evidence-based, culturally adaptable approach for suicide prevention in conflict-affected youth populations.

## Figures and Tables

**Table 1:** Demographic and Clinical Characteristics of the Sample (N = 900).

Demographic					(%)
Gender (female)					(61.1%)
Marital status					
Single					(66.7%)
Married					(23.1%)
Divorced/Separated					(7.9%)
Other					(2.3%)
Field of study					
Humanities					(23.3%)
Social sciences					(25.6%)
Natural sciences					(20.0%)
Engineering					(16.7%)
Health Sciences					(14.4%)
	Mean	SD	Min	Max	
Age	24.91	6.39	18	31	
Clinical Characteristics					
Depression	9.66	6.1	0	27	
Anxiety	7.67	5.26	0	21	
Resilience	17.96	4.06	6	30	
Self-Efficacy	15.85	7.17	0	30	
Attitude Toward Help-Seeking	24.91	7.39	14	60	
12 months of suicidal ideation					(8.8%)
Moderate-Severe Symptoms of Depression					(42.3%)
Moderate-Severe symptoms of Anxiety					(33.9%)

**Note.** Depression and anxiety were measured using PHQ-9 and GAD-7, respectively. Resilience = Brief Resilience Scale; Self-Efficacy = General Self-Efficacy Scale. Help-seeking = ATSPPH-SF. “Moderate–Severe” cutoffs are defined as a PHQ-9 score of 10 or higher and a GAD-7 score of 10 or higher. Suicidal ideation based on past 12-month reports using the Columbia–Suicide Severity Rating Scale (C-SSRS). SD = standard deviation; Min = minimum; Max = maximum.

**Table 2:** Group differences between participants with and without suicidal ideation in the past 12 months.

Variable	Suicidal Ideation (*n* = 87)	Non-Suicidal Ideation (*n* = 812)	Test Statistic	*p*	Cohen’s d	95% CI (Lower)	95% CI (Upper)
Age (M, SD)	24.59 (7.48)	24.51 (6.29)	t = 0.59	0.610	0.060	−0.171	0.291
Marital Status							
Single	(9.2%)	(66.6%)	χ^2^ = 3.98	0.264			
Married	(6.7%)	(23.9%)					
Divorced/Separated	(8.5%)	(8.0%)					
Other	(18.8%)	(2.0%)					
Field of Study							
Humanities	(26.4%)	(23.0%)	χ^2^ = 1.48	0.831			
Social Sciences	(27.6%)	(25.4%)					
Natural Sciences	(16.1%)	(20.4%)					
Engineering	(14.9%)	(16.9%)					
Health Sciences	(14.9%)	(14.4%)					
Gender, Female (%)	(50.6%)	(62.1%)	χ^2^ = 4.00	0.055			
Depression	13.87 (6.22)	9.25 (6.13)	t = −4.18	<0.001	−0.721	−10.113	−0.391
Anxiety	11.35 (5.27)	7.31 (5.11)	t = −6.67	<0.001	−0.787	−10.020	−0.553
Resilience	16.56 (4.54)	18.09 (3.89)	t = 3.35	<0.001	0.395	0.163	0.636
Self-efficacy	11.51 (6.50)	16.26 (7.09)	t = 5.71	<0.001	0.621	0.044	0.906
Attitude Toward Help-Seeking	15.56 (5.89)	18.90 (7.01)	t = 3.91	0.002	0.211	−0.012	0.421

**Table 3:** Logistic regression analysis examining factors associated with suicidal ideation in the past 12 months.

Predictor	B (SE)	Wald χ^2^	*p*	Odds Ratio [95% CI]
Depression	0.07 (0.02)	8.21	0.004	1.07 [1.02, 1.13]
Anxiety	0.10 (0.03)	10.32	0.001	1.11 [1.04, 1.19]
Resilience	−0.01 (0.02)	0.24	0.620	0.99 [0.94, 1.05]
Self-Efficacy	−0.06 (0.02)	10.89	0.001	0.94 [0.91, 0.97]
Help-Seeking	0.01 (0.01)	1.21	0.270	1.01 [0.98, 1.04]
Age	0.00 (0.01)	0.11	0.740	1.00 [0.98, 1.03]
Gender	0.14 (0.12)	1.58	0.210	1.15 [0.90, 1.47]

**Note**. Logistic regression model predicting 12-month suicidal ideation. Significant predictors are in bold. Gender coded: 0 = male, 1 = female. Hosmer–Lemeshow goodness-of-fit: χ^2^_(8)_ = 6.72, *p* = 0.57. OR = odds ratio; CI = confidence interval.

**Table 4:** Moderated Mediation Models for Depression and Anxiety Predicting Suicidal Ideation.

Predictor	Pathway	Effect	SE	95% CI	*p*-Value
Depression	Direct effect on suicidal ideation	0.038	0.005	[0.029, 0.047]	<0.001
	Indirect effect via self-efficacy	0.003	0.001	[0.001, 0.005]	0.002
	Index of moderated mediation (Resilience)	0.0001	0.0001	[−0.0001, 0.0003]	ns
Anxiety	Direct effect on suicidal ideation	0.039	0.005	[0.029, 0.048]	<0.001
	Indirect effect via self-efficacy	0.0036	0.0014	[0.0012, 0.0066]	0.003
	Index of moderated mediation (Resilience)	0.0001	0.0001	[−0.0001, 0.0003]	ns

**Note**. Effect estimates are based on PROCESS Model 14 with 5000 bootstrap samples. Indirect effects are mediated by self-efficacy, and resilience is modeled as a moderator of the indirect effect. CI = confidence interval; SE = standard error; ns = not significant.

## Data Availability

The data supporting the findings of this study are available from the corresponding author upon reasonable request.
